# VoxMore: technological artifact to assist voice acoustic evaluation in the teaching-learning process and clinical practice

**DOI:** 10.1590/2317-1782/20232022166en

**Published:** 2023-10-30

**Authors:** Samuel Ribeiro de Abreu, Ronei Marcos de Moraes, Perla do Nascimento Martins, Leonardo Wanderley Lopes

**Affiliations:** 1 Universidade Federal da Paraíba - UFPB - João Pessoa (PB), Brasil.; 2 Universidade Estadual do Centro-Oeste - UNICENTRO - Irati (PR), Brasil.

**Keywords:** Voice, Assessment, Acoustic, Teaching, Technology

## Abstract

**Purpose:**

to present a technological artifact, the VoxMore plugin, to assist the academic teaching of voice acoustic assessment, as well as to optimize the speech therapy intervention in the practice of vocal clinics.

**Methods:**

this is a multidisciplinary methodological study for the development of a technological artifact, a plugin, to be used in the Praat software. This tool performs vocal acoustic analysis and generates a report, with information and images referring to the domains of time, frequency, time-frequency, and que-frequency, as well as values of acoustic measures related to fundamental frequency (f0), period measures, disturbance measures of the period of f0, f0 amplitude perturbation measurements, spectral measurements, glottal noise measurements and cepstral measurements.

**Results:**

in the VoxMore acoustic report, four files are generated with the following information: oscillograms of the voice signal and traces of f0 and intensity; images related to the frequency domain, Fourier spectrum and LPC spectrum, and to the time-frequency domain, spectrogram; information on cepstral and cepstrogram analysis; the values of all acoustic measurements, in numerical results format and in vertical bar graphs.

**Conclusion:**

VoxMore can contribute both to the teaching-learning process, acting as an auxiliary tool with a formative character in the undergraduate and graduate courses in Speech-Language Pathology, as well as to the clinical practice process, making the use of acoustic analysis in the vocal clinic feasible and supporting decision-making by speech-language pathologist.

## INTRODUCTION

Vocal disorder assessment, diagnosis, and monitoring should preferably involve multidimensional data, such as laryngeal imaging, auditory-perceptual assessment, acoustic analysis, aerodynamic assessment, and the patient’s self-assessment^(1)^.

In the context of multidimensional voice assessment, acoustic analysis is a support tool that helps characterize and quantify voice quality deviations more objectively^(2)^. In clinical practice, acoustic analysis is one of the main methods to assess and monitor voice disorders^(3)^. It involves voice signal digital processing techniques, enabling the characterization and extraction of acoustic measures of the signal in the time, frequency, time-frequency, and quefrency domains^(4)^.

Acoustic measures can be associated with auditory-perceptual voice quality assessment^(5)^, laryngeal imaging data^(6)^, and aerodynamic voice production measures^(1)^. Combining these data, along with the patient’s self-assessment, helps clinicians understand systemic and/or specific voice disorder manifestations^(3)^.

However, speech-language-hearing (SLH) students often do not understand the relevance of acoustic analysis to clinical practice^(7)^. Moreover, basic knowledge of acoustic analysis of the voice is often not retained in the transition from undergraduate to postgraduate studies^(8)^. Using acoustic analysis in clinical practice during undergraduate training supports auditory-perceptual assessment and is an important task in voice assessment clinical training^(3)^.

Acoustic assessment has been widely discussed in the literature^(4)^. Studies report indices with robust multiparametric measures, such as the Acoustic Vocal Quality Index (AVQI)^(9)^ and Acoustic Breathiness Index (ABI)^(10)^, which are used as scripts in Praat. Despite these studies, there is yet no international standardization of the most important acoustic measures to study. Moreover, there is little evidence-based pedagogical instruction to teach about the applicability of acoustic analyses in vocal assessment.

Hence, some questions are raised: How can we create resources that effectively contribute to teaching acoustic analysis? What characteristics must a technological resource have to help students learn acoustic analysis? How can we develop or use software that meets the specificities of the knowledge about acoustic analysis in vocal clinical practice?

Given the scarcity of evidence on effectively teaching the topic, the answers to these questions are not trivial. Problem-based learning helped by technologies^(11)^ can be an attractive way to help maximize the students’ learning, interest, and dedication in classes, making them protagonists of their knowledge construction, and helping them develop critical and reflexive reasoning to solve problems.

Praat has been widely used in vocal acoustic analysis to digitally process the voice. This free software allows users to develop scripts, create plugins, and operate in connection with other programming languages^(12)^.

Given the above, this study aimed to present a technological resource - a Praat plugin named VoxMore - to help teach acoustic analysis of the voice to undergraduate and postgraduate SLH students and optimize the use of acoustic analysis in vocal clinical practice.

## METHODS

This methodological multidisciplinary study aimed to develop a technological resource for the acoustic analysis of the voice. The development team had a SLH therapist, an engineer, and a statistician.

This study was assessed and approved by the originating institution’s Research Ethics Committee (number 52492/12). Research participants were dysphonic and vocally healthy subjects of both sexes, aged 19 to 60 years, treated at the originating institution’s voice laboratory. All participants signed an informed consent form, authorizing their participation in the research.

The voice samples were recorded in Fonoview software, version 4.5, CTS Informática, in an all-in-one Dell desktop, unidirectional cardioid microphone manufactured by Sennheiser, model E-835, placed on a pedestal and attached to a Behringer preamplifier, model U-Phoria UMC 204. The voices were collected in an acoustically treated recording booth, with noise under 50 dB SPL, 44100-Hz sampling rate, 16 bits per sample, with the microphone placed 10 cm away from the patient’s mouth. To collect their voices, patients were standing, with the pedestal in front of them, at the said distance between their mouths and the microphone.

The resource was developed for Praat, version 6.2.10, and can be used in this and later versions, generating reports with images and acoustic measure values.

The images are shown in the time domain, with oscillograms; frequency domain, with Fourier spectrum and linear prediction coding (LPC); time-frequency domain, with narrowband spectrogram; and quefrency domain, with cepstral peak prominence (CPP), cepstral peak prominence-smoothed (CPPS), and cepstrogram. The following taxonomy was used to group acoustic measures: f0 measures, period measures, f0 period perturbation measures, f0 amplitude perturbation measures, spectral measures, glottal noise measures, and CPPS cepstral measures.

In the current VoxMore version, v1.0.0, the plugin is applied to acoustic analyses of sustained vowels. The voice sample used in this study to demonstrate the results was the sustained vowel /Ɛ/.

The maximum analysis period for each voice signal was set at 3 seconds. If any recording lasted more than 3 seconds, the plugin analyzes the 3 central seconds of the recording. If the emission is shorter than 3 seconds, the plugin analyzes the whole recording. However, signals whose emission lasts less than 1 second cannot be feasibly assessed for an effective analysis. The sound signal time was set based on studies that used AVQI^(9)^ and ABI^(10)^.

The first step to creating this resource was developing the plugin structure. Preliminary versions were created in the process, during which the development team reached a consensus on the information it would present, the acoustic measures to be applied, and the report format, which was conceived in a logical sequence of information, with a structure adapted from the literature^(13)^. These measures were selected considering that some of them are classified as traditional measures^(4)^, also applied in the AVQI^(9)^ and ABI^(10)^ research and others are part of a meta-analysis study^(4)^.

In the time domain, the report has four oscillogram images of the voice signal: complete signal, with a maximum of 3 seconds; signal of the initial 50 milliseconds; signal of the central 50 milliseconds; and signal of the final 50 milliseconds. Another two images are also shown, related to the f0 tracing and signal intensity in relation to the total time.

In the frequency domain, it presents six images of the soundwave Fourier spectrum, sampled at 44100 Hz: spectrum image up to 22.05 kHz, the whole frequency band; spectrum image up to 11 kHz; and an image with the Fourier spectrum and LPC spectrum at 5.5 kHz. The other three images are of lower frequency bands, one up to 2.75 kHz, another up to 1.38 kHz, and the last one up to 690 Hz, to better visualize the harmonics.

Concerning the time-frequency domain, it presents one image of a narrowband spectrogram. In the quefrency domain, the images are of CPP, CPPS, and the cepstrogram.

Lastly, the report presents all acoustic measures in numerical results and vertical bar charts:

- f0 measures: mean, standard deviation, first quartile, median, third quartile, minimum, maximum, and variation coefficient.
- Period measures: mean, standard deviation (PSD), and natural logarithm of the standard deviation (NLPSD).
- f0 period perturbation measures: jitter (local), jitter (local, absolute), jitter (RAP), jitter (PPQ5), and jitter (DDP).
- f0 amplitude perturbation measures: shimmer (local), shimmer (dB), shimmer (APQ3), shimmer (APQ5), shimmer (APQ11), and shimmer (DDA).
- Spectral measures: harmonics-to-noise ratio (HNR), the harmonics-to-noise ratio of Dejonckere (HNRD), the relative level of high-frequency noise energy (HFNO), the amplitude difference between the first and second harmonics (H1H2), decline, and tilt.
- Glottal noise measures: glottal noise excitation at bandwidths of 1000, 2000, and 3000 Hz.
- CPPS cepstral measures: mean, standard deviation, first quartile, median, third quartile, minimum, maximum, variation coefficient, and CPPS f0.

The following measures, available in Praat, were also included: intensity, mean correlation, voice breaks, and unvoiced frames. The mean correlation was included because it is a similar measure to Pearson’s r at the autocorrelation peak, described in meta-analysis studies^(4,6)^. Voice breaks and unvoiced frames were included because the development team has been using these measures to identify voice breaks and unvoiced frames in the sound signal. It is important to highlight that threshold configurations for some measures were adapted, as seen in other studies^(9,10)^.

The header of the acoustic report contains the patient’s data, collected with a form in which they informed their name, sex, age, occupation, auditory-perceptual assessment result on the GRBAS and CAPE-V scales, and the examiner’s identification. The date of the acoustic report is automatically inserted in the header. After filling out the form, a screen appears to the user, in which they select the folder where the file will be saved. Then, it will generate a report with four files. If the operating system is Windows, the files will be in PNG format; if it is Linux or Mac, they will be in PDF format.

VoxMore is installed by downloading the file available on the internet^(14)^. A compressed file is downloaded, which must be uncompressed in the preferred Praat folder. Locating this folder depends on the computer’s operating system, as each one uses different paths, as described below (consider user_name as the one used in the operating system):

- Linux: /home/user_name/.praat-dir
- Mac: /Users/user_name/Library/Preferences/Praat Prefs/
- Windows: C:\Users\user_name\Praat

If Praat is open when the plugin is installed, Praat will have to be closed and reopened for the VoxMore installation to work. After finishing the installation, VoxMore will be available from Praat’s dynamic menu.

## RESULTS

VoxMore use was exemplified in this study with the emission of a sustained /Ɛ/ vowel by a dysphonic female, 36 years old, with nodules and an hourglass-shaped gap.

**Figure 1:**
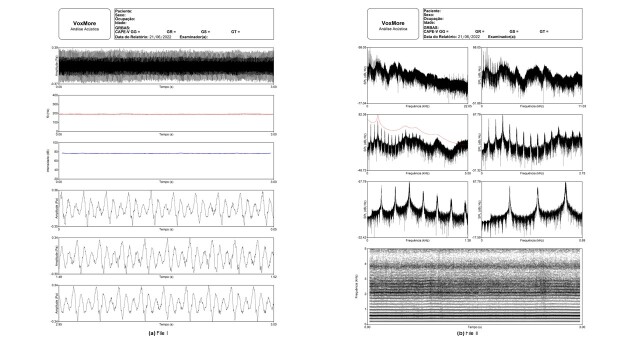
(a) File I: Oscillogram information on the voice signal, f0 tracing, and intensity tracing. (b) File II: information on the frequency domain, Fourier spectrum, and LPC spectrum, and time-frequency domain, spectrogram

Figure 1a shows six images of the acoustic report generated by VoxMore - four oscillograms of the voice signal, one f0 tracing, and one intensity tracing. Figure 1b shows seven images - six of the frequency domain and one of the time-frequency domain. The time-frequency image provides information on the narrowband spectrogram.

**Caption:** CAPE-V: GG = Overall severity/grade; GR = Roughness grade; GS = Breathiness grade; GT = Strain grade; (a) File I; (b) File II

**Figure 2:**
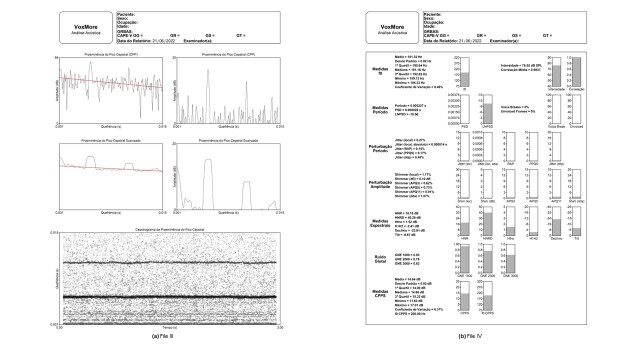
(a) File III: information on the cepstral analysis and cepstrogram. (b) File IV: information on the acoustic measures

Figure 2a shows five images, with cepstral analysis information. The first two images show the cepstral peaks with the regression line and the cepstral peaks subtracted from the regression line. The third and fourth images respectively show smoothed cepstral peaks with the regression line and smoothed cepstral peaks subtracted from the regression line. The last image shows the cepstrogram, with the time and quefrency domains, both in seconds. Figure 2b shows all acoustic measure values as numerical results and vertical bar charts to help visualize and understand the information.

**Caption:** CAPE-V: GG = Overall severity/grade; GR = Roughness grade; GS = Breathiness grade; GT = Strain grade; (a) File III; (b) File IV

## DISCUSSION

The dynamics between the university and the teaching clinic and optimized clinical practice highlighted the need for new strategies to train vocal acoustic assessment. Hence, VoxMore provides a better understanding of acoustic information and helps professors, students, and SLH therapists explore this analysis in various domains shown in the report.

In the time domain, oscillograms can reinforce concepts related to period, intensity, and f0. Segmented oscillograms of voices with moderate to intense deviations show period and amplitude perturbations, approaching concepts of jitter and shimmer. The intensity and f0 tracings make it possible to correlate concepts related to the perception of loudness and pitch and observe possible instabilities during the emission.

In the frequency domain, images of the Fourier transform spectrum and LPC spectrum can be analyzed, making it possible to explore concepts on source-filter, harmonics, formants, spectral decline, and so on. Concerning the time-frequency domain, the spectrographic analysis protocol can be applied to the narrowband spectrogram to analyze acoustic information^(15)^.

The analysis of the cepstral peak images can reinforce more clearly CPP and CPPS concepts, considering the difference between the cepstral peak prominence and the regression line and the concept of smoothing this peak. This study suggests that the cepstrogram be used as a new approach in assessments and that further studies verify the information that can be extracted from the cepstrogram.

The report images favor both educational and professional activities, providing visual information that can be analyzed and compared in different vocal qualities.

Regarding the description of acoustic measures, one of the main benefits of VoxMore is that it encompasses various measures that can be correlated with physical acoustic concepts and analyzed quantitatively, considering the deviation in the sound signal and comparing it with the normative data^(2)^, and qualitatively, considering the physiological mechanisms related to phonation and possible associated voice disorders. These data can help understand the auditory-perceptual and laryngeal assessments, which has posed a challenge to both clinicians and researchers^(16)^, and help follow up on therapy progress.

A limitation of this study is that the plugin is being experimentally tested in only one university. Thus, as soon as it is made available for public use, VoxMore is expected to be tested in other populations and contexts, which may provide suggestions for possible improvements in the plugin.

## CONCLUSION

VoxMore plugin is a technological resource developed to stimulate students’ skills in their training and provide an instrument to practicing professionals. Hence, VoxMore may contribute to both teaching-learning, as an auxiliary training tool in undergraduate and postgraduate SLH courses, and clinical practice, making feasible the use of acoustic analysis in vocal clinical practice, and helping SLH therapists in decision-making.
