# Supplementary material for: VoxMore: technological artifact to assist voice acoustic evaluation in the teaching-learning process and clinical practice
Source: Codas. 2023 Oct 30;35(6):e20220166. doi: 10.1590/2317-1782/20232022166en (PMC10702702; doi:10.1590/2317-1782/20232022166en)
Supplement: Translation

# Translation: VoxMore: artefato tecnológico para auxiliar a avaliação acústica da voz no processo ensino-aprendizagem e prática clínica

**DOI:** 10.1590/2317-1782/20232022166pt

**Authors:** Samuel Ribeiro de Abreu, Ronei Marcos de Moraes, Perla do Nascimento Martins, Leonardo Wanderley Lopes

**Affiliations:** 1 Universidade Federal da Paraíba - UFPB - João Pessoa (PB), Brasil.; 2 Universidade Estadual do Centro-Oeste - UNICENTRO - Irati (PR), Brasil.

**Keywords:** Voz, Avaliação, Acústica, Ensino, Tecnologia

## Abstract

**Objetivo:**

apresentar um artefato tecnológico, o plugin VoxMore, para auxiliar o ensino acadêmico da avaliação acústica da voz, bem como otimizar a intervenção fonoaudiológica na prática da clínica vocal.

**Método:**

trata-se de um estudo metodológico de caráter multidisciplinar para o desenvolvimento de um artefato tecnológico, plugin, a ser utilizado no software Praat. Essa ferramenta realiza análise acústica vocal e gera um relatório, com informações e imagens referentes aos domínios do tempo, frequência, tempo-frequência, e quefrência, bem como valores de medidas acústicas relacionadas a frequência fundamental (f0), medidas de período, medidas de perturbação do período da f0, medidas de perturbação de amplitude da f0, medidas espectrais, medidas de ruído glotal e medidas cepstrais.

**Resultados:**

no relatório acústico do VoxMore são gerados quatro arquivos com as seguintes informações: oscilogramas do sinal de voz e traçados da f0 e intensidade; imagens relacionadas ao domínio da frequência, espectro de Fourier e espectro de LPC, e ao domínio do tempo-frequência, espectrograma; informações sobre a análise cepstral e cepstrograma; valores de todas as medidas acústicas, no formato de resultados numéricos e em gráficos de barras verticais.

**Conclusão:**

o VoxMore pode contribuir tanto com o processo de ensino-aprendizagem, atuando como ferramenta auxiliar com caráter formativo nas disciplinas de graduação e pós-graduação em Fonoaudiologia, quanto com o processo da prática clínica, tornando viável a utilização da análise acústica na clínica vocal e apoiando a tomada de decisão dos fonoaudiólogos.

## INTRODUÇÃO

Avaliação, diagnóstico e monitoramento de distúrbios vocais devem envolver, preferencialmente, informações multidimensionais, como o exame visual laríngeo, julgamento perceptivo-auditivo, análise acústica, avaliação aerodinâmica e autoavaliação do paciente^(1)^.

No universo da avaliação multidimensional da voz, a análise acústica é uma ferramenta de apoio que auxilia caracterizar e quantificar de maneira mais objetiva o desvio da qualidade vocal^(2)^. No contexto clínico, a análise acústica está entre os principais métodos de avaliação e monitoramento dos distúrbios da voz^(3)^. Essa análise envolve técnicas de processamento digital do sinal de voz, com possibilidade de caracterização e extração de medidas acústicas do sinal, seja no domínio do tempo, no domínio da frequência, no domínio do tempo-frequência, ou no domínio da quefrência^(4)^.

As medidas acústicas podem ser associadas ao julgamento perceptivo-auditivo da qualidade vocal^(5)^, aos dados do exame visual laríngeo^(6)^ e às medidas aerodinâmicas da produção vocal^(1)^. A integração desses dados, juntamente com a autoavaliação do paciente, permite ao clínico compreender de maneira sistêmica, e/ou específica, a manifestação de um distúrbio de voz^(3)^.

No entanto, os estudantes de Fonoaudiologia muitas vezes não conseguem compreender a relevância da análise acústica aplicada à prática clínica^(7)^. Além de não compreender a importância dessa análise, o conhecimento básico da análise acústica da voz muitas vezes não é mantido na transição entre a graduação e a pós-graduação^(8)^. A aplicação da análise acústica na prática clínica, durante a graduação, é um caminho para apoiar a avaliação perceptivo-auditiva, como também uma importante tarefa do treinamento clínico em avaliação vocal^(3)^.

A avaliação acústica tem sido amplamente discutida na literatura^(4)^. Pesquisas referem índices compostos por medidas multiparamétricas que são considerados robustos, como Acoustic Vocal Quality Index (AVQI)^(9)^ e Acoustic Breathiness Index (ABI)^(10)^, inclusive utilizados como *script* do Praat. Apesar desses estudos, ainda não existe uma padronização internacional de quais medidas acústicas são as mais importantes a serem estudadas. Também observa-se pouca orientação pedagógica baseada em evidências para o ensino sobre a aplicabilidade da análise acústica em uma avaliação vocal.

Diante disso, surgem alguns questionamentos: como criar recursos que contribuam efetivamente para a atividade docente em análise acústica? Quais características um artefato tecnológico deve possuir para auxiliar a aprendizagem do estudante em análise acústica? Como desenvolver ou aproveitar um *software* que atenda às especificidades dos conhecimentos da análise acústica na clínica vocal?

Dada a escassez de evidências sobre o ensino efetivo do tema, as respostas a esses questionamentos não são triviais. A aprendizagem baseada em problemas, com o auxílio de tecnologias^(11)^, pode ser uma maneira atrativa de contribuir para a maximização do aprendizado, interesse e empenho dos discentes nas aulas, tornando-os protagonistas na construção do seu conhecimento, bem como desenvolver raciocínio crítico e reflexivo para resolução de problemas.

Na análise acústica vocal, o Praat tem sido amplamente utilizado em aplicações de processamento digital de voz, por ser um *software* gratuito, que permite ao usuário desenvolver *scripts*, criação de *plugins* e possibilita uma interoperabilidade com outras linguagens de programação^(12)^.

Diante do exposto, este estudo teve por objetivo apresentar um artefato tecnológico, um *plugin* do Praat denominado VoxMore, para auxiliar o ensino da análise acústica da voz, visando estudantes de graduação e pós-graduação em Fonoaudiologia, bem como otimizar o uso da análise acústica na clínica vocal.

## MÉTODO

Trata-se de um estudo metodológico de caráter multidisciplinar para o desenvolvimento de um artefato tecnológico para análise acústica da voz. A equipe desenvolvedora foi composta por um fonoaudiólogo, um engenheiro e um estatístico.

Este trabalho foi avaliado e aprovado pelo comitê de Ética em Pesquisa da Instituição de origem (número 52492/12). Participaram dessa pesquisa sujeitos vocalmente saudáveis e disfônicos, ambos os gêneros, faixa etária de 19 a 60 anos, atendidos no laboratório de voz da Instituição de origem. Todos os participantes assinaram o Termo de Consentimento Livre e Esclarecido, autorizando a participação na pesquisa.

Para a gravação das amostras vocais foi utilizado o *software* Fonoview, versão 4.5, CTS Informática, *desktop* Dell all-in-one, microfone cardioide unidirecional, marca Sennheiser, modelo E-835, localizado em um pedestal e acoplado a um pré-amplificador Behringer, modelo U-Phoria UMC 204. As vozes foram coletadas em cabine de gravação com tratamento acústico, ruído inferior a 50 dB NPS, taxa de amostragem de 44.100 Hz, com 16 bits por amostra e distância de 10 cm entre o microfone e a boca do paciente. Para a coleta das vozes, os pacientes estavam em pé, situando-se o pedestal à sua frente, de acordo com a distância preconizada entre a boca e o microfone.

O recurso foi desenvolvido para o *software* Praat, versão 6.2.10, podendo ser utilizado nessa versão e posteriores, gerando um relatório com imagens e valores de medidas acústicas.

As imagens são apresentadas no domínio do tempo, através de oscilogramas; no domínio da frequência, através do espectro de Fourier e dos coeficientes de predição linear (LPC); no domínio do tempo-frequência, através do espectrograma de banda estreita; e no domínio da quefrência, através do pico cepstral proeminente (CPP) e pico cepstral proeminente suavizado (CPPS) e cepstrograma. Foi adotada a seguinte taxonomia para agrupar as medidas acústicas: medidas da f0, medidas de período, medidas de perturbação do período da f0, medidas de perturbação de amplitude da f0, medidas espectrais, medidas de ruído glotal e medidas cepstrais do CPPS.

Na atual versão do VoxMore, v1.0.0, o *plugin* é aplicado à análise acústica de vogais sustentadas. No presente estudo, a amostra vocal utilizada para demonstração dos resultados foi a vogal /Ɛ/ sustentada.

Foi fixado um período máximo de análise de três segundos para cada sinal de voz. Caso uma gravação seja maior do que três segundos, o *plugin* vai analisar os três segundos centrais da gravação. Caso seja uma emissão menor do que três segundos, o *plugin* vai analisar toda a gravação. É válido ressaltar que sinais com tempo de emissão inferior a um segundo não são viáveis para realizar uma análise efetiva. O tempo do sinal sonoro foi estabelecido com base nos trabalhos que utilizam os índices AVQI^(9)^ e ABI^(10)^.

O primeiro passo para a criação desse recurso foi o desenvolvimento da estrutura do *plugin*. Durante o período de criação foram realizadas versões preliminares e, durante o processo de criação, a equipe desenvolvedora entrou em consenso sobre as informações a serem apresentadas, as medidas acústicas aplicadas e o formato do relatório, o qual foi idealizado em uma sequência lógica de informações, sendo essa estrutura adaptada com base na literatura^(13)^. Essas medidas foram selecionadas, considerando que algumas são classificadas como medidas tradicionais^(4)^, medidas aplicadas nas pesquisas dos índices AVQI^(9)^ e ABI^(10)^, e outras fazem parte de um estudo de metanálise^(4)^.

No domínio do tempo, o relatório apresenta quatro imagens de oscilogramas do sinal de voz: sinal completo, máximo três segundos; sinal dos 50 milissegundos iniciais; sinal dos 50 milissegundos centrais, e sinal dos 50 milissegundos finais. Além dessas imagens, também são apresentadas outras duas imagens, relacionadas aos traçados da f0 e da intensidade do sinal, em relação ao tempo total.

No domínio da frequência são apresentadas seis imagens do espectro de Fourier da onda sonora, amostrada em 44.100 Hz: imagem do espectro até 22,05 kHz, toda a faixa de frequência; imagem de um espectro até 11 kHz; e imagem contendo o espectro de Fourier e o espectro de LPC na faixa de 5,5 kHz. As outras três imagens são de faixas de frequência menores, sendo uma até 2,75 kHz, outra até 1,38 kHz e uma última até 690 Hz, utilizadas para uma melhor visualização dos harmônicos.

Em relação ao domínio do tempo-frequência é apresentada uma imagem de um espectrograma de banda estreita. No domínio da quefrência, as imagens exibidas são do CPP, CPPS e o cepstrograma.

Por fim, o relatório os valores de todas as medidas acústicas, no formato de resultados numéricos e em gráficos de barras verticais:

- Medidas da f0: média, desvio padrão, primeiro quartil, mediana, terceiro quartil, mínimo, máximo e coeficiente de variação.
- Medidas do período: média, desvio padrão (PSD) e logaritmo natural do desvio padrão (LNPSD).
- Medidas de perturbação do período da f0: *jitter* (local), *jitter* (local, absoluto), *jitter* (RAP), *jitter* (PPQ5) e *jitter* (ddp).
- Medidas de perturbação de amplitude da f0: *shimmer* (local), *shimmer* (dB), *shimmer* (APQ3), *shimmer* (APQ5), *shimmer* (APQ11) e *shimmer* (dda).
- Medidas espectrais: proporção harmônico-ruído (HNR), proporção harmônico-ruído de Dejonckere (HNRD), nível relativo de energia do ruído de alta frequência (Hfno), diferença entre a amplitude do primeiro e o segundo harmônico (H1H2), declínio e *tilt*.
- Medidas de ruído glotal: excitação do ruído glotal em larguras de banda de 1.000 Hz, 2.000 Hz e 3.000 Hz.
- Medidas cepstrais do CPPS: média, desvio padrão, primeiro quartil, mediana, terceiro quartil, mínimo, máximo, coeficiente de variação e f0 do CPPS.

Também foram inseridas outras medidas disponíveis no Praat, sendo intensidade, correlação média, *voice breaks* e *unvoiced frames*. A correlação média foi inserida pois é uma medida semelhante à medida *pearson r at autocorrelation peak,* descrita em estudos de metanálises^(4,6)^. As medidas *voice breaks* e *unvoiced frames* foram inseridas pois a equipe desenvolvedora tem utilizado tais medidas para a identificação de quebras vocais e de trechos não vozeados no sinal sonoro. Ressalta-se que configurações de limiares, de algumas medidas, foram adaptadas conforme observado em outros estudos^(9,10)^.

Em relação ao relatório acústico, para compor o cabeçalho com as informações do paciente, foi criado um formulário no qual o usuário deve inserir o nome do paciente, sexo, idade, ocupação, resultado da avaliação perceptivo-auditiva nas escalas GRBAS e CAPE-V, e identificação do examinador. A data do relatório acústico é inserida automaticamente no cabeçalho. Após o preenchimento do formulário, irá aparecer uma tela para o usuário, na qual ele deve selecionar a pasta para salvar o documento. Realizadas essas ações, será gerado um relatório composto por quatro arquivos. Se o sistema operacional for Windows, os arquivos serão no formato PNG, se for Linux ou Mac, os arquivos serão no formato PDF.

Para instalar o VoxMore, o *download* do arquivo deve ser feito pelo *link* disponível na internet^(14)^. Será baixado um arquivo compactado, o qual deve ser descompactado na pasta de preferências do Praat. Para localizar essa pasta, deve ser observado o sistema operacional utilizado no computador, pois cada sistema operacional utiliza caminhos diferentes, conforme descrição abaixo (considerar user_name como nome do usuário utilizado no sistema operacional):

- Linux: /home/user_name/.praat-dir
- Mac: /Users/user_name/Library/Preferences/Praat Prefs/
- Windows: C:\Users\user_name\Praat

Vale ressaltar que, se o Praat estiver aberto no momento da instalação do *plugin*, será necessário encerrar e iniciar novamente o Praat para que a instalação do VoxMore funcione. Após a instalação concluída, o VoxMore estará disponível para ser acessado no menu dinâmico do Praat.

## RESULTADOS

Para exemplificar a utilização do VoxMore, no presente estudo foi utilizada a emissão de vogal /Ɛ/ sustentada, voz disfônica, com presença de nódulos e fenda em ampulheta, sexo feminino, idade 36 anos.

Em relação ao relatório acústico gerado pelo VoxMore, a Figura 1a apresenta seis imagens, sendo quatro imagens de oscilogramas do sinal de voz, uma do traçado da f0 e uma referente ao traçado da intensidade. Na Figura 1b são apresentadas sete imagens, sendo seis relacionadas ao domínio da frequência e uma ao domínio do tempo-frequência. A imagem relacionada ao domínio do tempo-frequência fornece informações sobre o espectrograma de banda estreita.

**Figura 1:** (a) Arquivo I: informações sobre oscilogramas do sinal de voz, traçado da f0 e traçado da intensidade. (b) Arquivo II: informações sobre domínio da frequência, espectro de Fourier e espectro de LPC, e domínio do tempo-frequência, espectrograma

**Legenda:** CAPE-V: GG = Grau geral do desvio; GR = Grau rugosidade; GS = Grau soprosidade; GT = Grau tensão; (a) Arquivo I; (b) Arquivo II

Já a Figura 2a apresenta cinco imagens contendo informações sobre a análise cepstral. As duas primeiras mostram os picos cepstrais com a reta de regressão e os picos cepstrais subtraídos da reta de regressão. Na sequência, terceira e quarta imagens mostram os picos cepstrais suavizados com a reta de regressão e os picos cepstrais suavizados subtraídos da reta de regressão, respectivamente. Na última imagem observa-se o cepstrograma, que tem o domínio do tempo e o domínio da quefrência, ambos em segundos. A Figura 2b apresenta os valores de todas as medidas acústicas, no formato de resultados numéricos e em gráficos de barras verticais, para facilitar a visualização e compreensão das informações.

**Figura 2:** (a) Arquivo III: informações sobre a análise cepstral e cepstrograma. (b) Arquivo IV: informações sobre as medidas acústicas

**Legenda:** CAPE-V: GG = Grau geral; GR = Grau rugosidade; GS = Grau soprosidade; GT = Grau tensão; (a) Arquivo III; (b) Arquivo IV

## DISCUSSÃO

Levando em consideração a dinamicidade entre a universidade e a clínica-escola, e a otimização de ações na prática clínica, foi observada a necessidade de novas estratégias para o treinamento da avaliação acústica vocal. Diante disso, o VoxMore possibilita uma melhor compreensão das informações acústicas, bem como condições para que docentes, alunos e fonoaudiólogos possam explorar tal análise em domínios variados, apresentados no relatório.

No domínio do tempo, os oscilogramas podem fortalecer conceitos relacionados ao período, intensidade e f0. Nos oscilogramas segmentados, em vozes que apresentam desvios moderados a intensos, é possível observar perturbações de período e amplitude, podendo ser abordados conceitos relacionados ao *jitter* e *shimmer*. Com os traçados da intensidade, e da f0, é possível correlacionar conceitos relacionados à percepção da *loudness* e *pitch*, bem como observar possíveis instabilidades durante a emissão.

No domínio da frequência é possível analisar imagens do espectro da transformada de Fourier e do espectro de LPC. Nesse contexto, podem ser explorados conceitos sobre fonte-filtro, harmônicos, formantes, declínio espectral, entre outros. Em relação ao domínio tempo-frequência, no espectrograma de banda estreita é possível aplicar o protocolo de análise espectrográfica para análise das informações acústicas^(15)^.

Na análise das imagens dos picos cepstrais podem ser reforçados, de forma mais clara, conceitos de CPP e CPPS, considerando a diferença do pico cepstral proeminente para a reta de regressão e o conceito de suavização desse pico. O presente estudo traz como sugestão que o cepstrograma seja utilizado como uma nova abordagem nas avaliações e, também, sugere que novos estudos sejam realizados, com o intuito de verificar as informações que podem ser extraídas do cepstrograma.

As imagens fornecidas pelo relatório favorecem tanto atividades educacionais quanto profissionais, pois fornecem informações visuais que podem ser analisadas e comparadas em diferentes qualidades vocais.

Sobre a descrição das medidas acústicas, um dos principais benefícios do VoxMore é abranger uma variedade de medidas que podem ser correlacionadas aos conceitos físicos da acústica e analisadas quantitativamente, considerando o desvio presente no sinal sonoro e comparação com dados normativos^(2)^, bem como podem ser analisadas qualitativamente, considerando os mecanismos fisiológicos relacionados à fonação e possíveis distúrbio vocais associados. Essas informações podem propiciar a compreensão da avaliação perceptivo-auditiva e da laríngea, condição essa que tem sido considerada um desafio tanto para clínicos quanto para pesquisadores^(16)^, bem como auxiliar no acompanhamento de uma evolução terapêutica.

Uma limitação considerada no presente estudo é a de que o *plugin* está sendo testado em apenas uma universidade, em caráter experimental. Sendo assim, logo que disponibilizado para uso público, espera-se que o VoxMore seja testado em outras populações, e contextos, que podem oferecer sugestões para possíveis melhorias no *plugin*.

## CONCLUSÃO

O *plugin* VoxMore é um artefato tecnológico desenvolvido para estimular as habilidades dos alunos em formação, bem como viabilizar a instrumentalização do profissional já formado. Nesse sentido, entende-se que o VoxMore pode contribuir tanto com o processo de ensino-aprendizagem, atuando como ferramenta auxiliar com caráter formativo nas disciplinas de graduação e pós-graduação em Fonoaudiologia, quanto com o processo da prática clínica, tornando viável a utilização da análise acústica na clínica vocal e apoiando a tomada de decisão dos fonoaudiólogos.
